# Expanding the scope of prehabilitation: reducing critical illness weakness across elective surgical patients scheduled for postoperative ICU care

**DOI:** 10.1186/s13054-023-04784-0

**Published:** 2024-01-02

**Authors:** Wen Gao, Jingfen Jin

**Affiliations:** 1 Hangzhou Normal University School of Nursing, Hangzhou, China; 2 Department of Nursing, Second Affiliated Hospital of Zhejiang University School of Medicine, Hangzhou, China

The article “Illness Weakness, Polyneuropathy and Myopathy: Diagnosis, treatment, and long-term outcomes” [1] sheds light on the pervasive issue of critical illness weakness (CIW) and provides a comprehensive overview of the diagnosis, treatment, and long-term outcomes of CIW in ICU settings. While the review meticulously addresses post-admission interventions and multidisciplinary approaches, it prompts a consideration of preventive strategies, specifically prehabilitation, for patients scheduled for postoperative ICU care.

Critical illness weakness profoundly impacts both the acute and long-term recovery of patients, and contributes to escalating healthcare costs [2]. While some postoperative patients experience temporary muscle weakness due to residual effects of neuromuscular blockade or inflammation, a notable proportion encounter persistent weakness. Notably, the incidence of enduring muscle weakness one year post-surgery can vary significantly, affecting 30% to 80% of surgical patients [3]. This variation underscores the complexity and seriousness of the condition, highlighting the need for more effective management and preventive strategies in the surgical care continuum. While early mobilization in the ICU has been shown to mitigate the risk of ICU-acquired weakness [4], it is crucial for patients anticipated to be admitted to the ICU with a preemptive intervention window to start interventions early. This strategy is not merely an early treatment post-CIW development but an essential proactive measure.

Prehabilitation, a series of preoperative interventions designed to improve patients' physical, psychological, and nutritional health, emerges as a critical preventive strategy [5]. The concept of prehabilitation is grounded in the belief that enhancing a patient's overall health status before surgery can lead to better postoperative outcomes. Patients may have a better chance of withstanding the stresses associated with surgery and critical care [6], for example, by retaining cardiopulmonary fitness and minimizing muscle loss, which is often linked to functional dependence [7, 8]. This approach is particularly relevant for elective surgery patients who have a scheduled surgery and, therefore, the opportunity to engage in rehabilitation measures.

Prehabilitation programs often include physical therapy and exercises designed to enhance muscle strength and endurance, which could be beneficial in mitigating the impact of prolonged bed rest and immobility in the ICU [9]. A recent systematic review reported that prehabilitation, involving targeted exercises and physical therapies, could prepare the patients’ musculature for the upcoming stress [10]. For cancer patients, prehabilitation could also attenuate the post-surgical lean body mass loss [11].

Multi-modal prehabilitation, extending beyond mere physical preparation, plays a crucial role in enhancing preoperative psychological readiness, significantly improving patients' mental well-being before undergoing surgery. Prehabilitation's psychological interventions, including counseling and stress-reduction techniques, help alleviate preoperative anxiety and depression, contributing to a more resilient mindset in patients [12]. Given the prevalence of cognitive disorders in ICU survivors, prehabilitation programs increasingly incorporate cognitive exercises. These exercises, including game playing, memory and recall training, and problem-solving tasks, have demonstrated effectiveness in enhancing cognitive reserve [13].

Prehabilitation also includes nutritional optimization as a key component, which could have a significant impact on reducing the risk or severity of CIW. Nutritional optimization before surgery is critical in enhancing a patient's overall health status and proper nutrition can help support protein anabolism, build and maintain muscle mass and strength [14], which is particularly important considering the muscle wasting often associated with ICU stays.

Despite these potential benefits, the challenges and limitations inherent in its implementation are set to become focal points for refinement. The diversity in patient health profiles, the variety in surgical procedures, and the differing ICU practices call for a more personalized approach to prehabilitation. This necessitates research designs that are flexible yet rigorous, capable of adapting to individual patient needs. Ensuring patient adherence to prehabilitation protocols, especially in the face of varying patient capacities and needs, will be a critical area of focus. Future research should aim to develop adaptable, inclusive prehabilitation strategies that not only respect but leverage the unique characteristics of each patient’s health and surgical journey, thus optimizing the role of prehabilitation in preventing and mitigating CIW.

Future research should focus on assessing the effectiveness of prehabilitation in preventing CIW among diverse elective surgery patients. This requires controlled trials that not only evaluate CIW in ICU settings but also explore long-term functional recovery. Such studies will enhance our understanding of prehabilitation's impact on CIW development and its sustained benefits.

## Data Availability

Not applicable.
